# Morroniside Attenuates Doxorubicin‐Induced Cardiotoxicity by Activating the PI3K/AKT/Nrf2/HO‐1 Pathway to Inhibit Ferroptosis and Oxidative Stress

**DOI:** 10.1002/jbt.71016

**Published:** 2026-07-27

**Authors:** Zhi‐Hui Lin, Xing‐Yu Lin, Wen‐Jie Lu, Meng‐Qi Wu, Dong‐Yan Song, Rui‐Dong Cheng

**Affiliations:** ^1^ Center for Rehabilitation Medicine, Department of Rehabilitation Medicine Zhejiang Provincial People's Hospital (Affiliated People's Hospital, Hangzhou Medical College), Zhejiang Engineering Research Center for Digital‐Intelligent Rehabilitation Equipment Hangzhou Zhejiang P. R. China; ^2^ Department of Cardiology, The Second Affiliated Hospital and Yuying Children's Hospital Wenzhou Medical University Wenzhou Zhejiang China; ^3^ School of Medicine, Shaoxing University Shaoxing Zhejiang China; ^4^ Department of Pediatrics, Zhejiang Provincial People's Hospital Center for Reproductive Medicine Hangzhou Zhejiang P.R. China; ^5^ Department of Cardiology the First Affiliated Hospital of Wenzhou Medical University Wenzhou Zhejiang China; ^6^ The First People's Hospital of Hangzhou Lin'an District, Affiliated Lin'an People's Hospital of Hangzhou Medical College Hangzhou Zhejiang China

**Keywords:** Dox‐induced cardiotoxicity, doxorubicin, ferroptosis, Morroniside, Nrf2, oxidative stress

## Abstract

Cardiotoxicity induced by doxorubicin (Dox) significantly contributes to increased mortality among cancer patients, yet available pharmacological interventions remain scarce. Recent studies suggest that ferroptosis is a key mechanism in the development of Dox‐induced cardiotoxicity (DIC). Morroniside (Mor), an active iridoid glycoside isolated from *Cornus officinalis*, exhibits multiple pharmacological properties such as antioxidant, anti‐ferroptotic, and anti‐inflammatory activities. Given this multi‐target profile, Mor shows potential as a treatment option for reducing DIC. This work was designed to examine the association between Mor and DIC. In vivo DIC model, C57BL/J mice received 5 mg/kg/d Mor via oral gavage for 5 weeks. In vitro DIC model, H9c2 cells were exposed to 10 μM Mor over a 48‐h period. Cardiac injury markers were quantified in serum and cell culture supernatants. Biochemical assays, western blotting, cellular immunofluorescence, and DHE/ROS staining were employed to evaluate ferroptosis and oxidative stress. Mor administration substantially reduced the levels of cardiac injury biomarkers while simultaneously attenuating ferroptosis and oxidative stress in vivo. In cellular models, Mor exhibited potent anti‐ferroptotic and antioxidant effects through Nrf2 pathway activation. Further mechanistic studies identified PI3K/AKT pathway as the upstream regulator of Nrf2 activation in response to Mor treatment. Our study provided the first evidence for Mor's cardioprotective effects against DIC. Mechanistically, Mor attenuated DIC by suppressing ferroptosis and reducing oxidative stress via activation of the PI3K/AKT/Nrf2/HO‐1 signaling pathway.

## Graphical abstract

Mor activates the PI3K/AKT pathway to promote Nrf2 nuclear translocation and HO‐1 expression, enhancing antioxidants, curbing Fe^2+^‐induced ROS/lipid peroxidation, suppressing ferroptosis, and alleviating Dox‐induced cardiotoxicity.

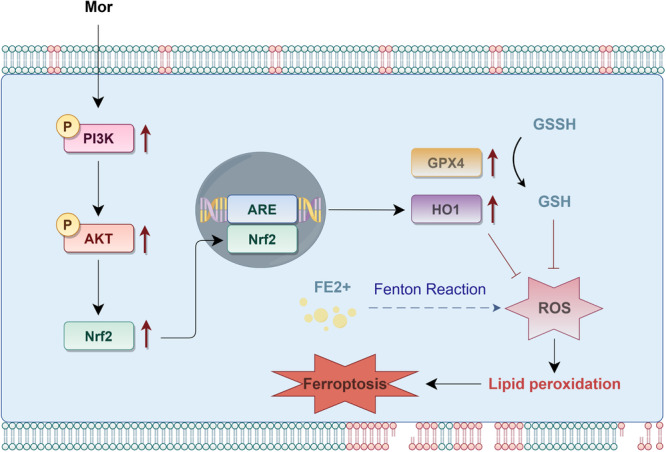

**Abbreviations**
CAT: catalase
CK‐MB: creatine Kinase‐MB
DIC: Dox‐induced cardiotoxicity
Doxorubicin: Dox
GPX4: glutathione peroxidase 4‌
GSH: glutathione
LDH: lactate dehydrogenase
MDA: malondialdehyde
Mor: morroniside
Nrf2: nuclear factor erythroid 2‐ related factor 2
ROS: reactive sxygen species
SOD: superoxide dismutase

## 1 Introduction

As a cornerstone anthracycline chemotherapeutic agent, doxorubicin (Dox) remains widely utilized in clinical oncology practice [1]. Nevertheless, Dox's non‐selective cytotoxicity leads to dose‐limiting cardiac damage, a condition clinically recognized as Dox‐induced cardiotoxicity (DIC) [2]. Despite the complex pathophysiology of DIC, excessive reactive oxygen species (ROS) production and resultant oxidative stress are widely recognized as key drivers of its progression [3, 4].

Ferroptosis, a recently discovered form of regulated cell death, is defined by iron‐dependent excessive lipid peroxidation [5]. Factors like iron, ROS, and membrane lipids mainly influence it [6]. Elevated intracellular Fe^2+^ levels promote ROS generation through the Fenton reaction, which subsequently induces lipid peroxidation in mitochondrial and cellular membranes [7, 8]. Recent studies further indicate that Dox disrupts cardiomyocyte iron homeostasis by promoting iron accumulation and impairing iron export, thereby creating a permissive environment for ferroptosis during DIC [9]. Moreover, mitochondrial injury has emerged as another critical event linking Dox exposure to ferroptosis: Dox‐induced mitochondrial dysfunction enhances ROS production, aggravates lipid peroxidation, and facilitates Fe2 + ‐dependent oxidative damage [10, 11]. Emerging evidence indicates ferroptosis may be the predominant cell death mechanism underlying DIC, surpassing other forms of programmed cell death in pathological significance [12]. Dox significantly downregulated key antioxidant defense components, most notably glutathione peroxidase 4 (GPX4) ‐ the key regulator of ferroptosis responsible for clearing lipid peroxides [13, 14]. Simultaneous decreases in superoxide dismutase (SOD) activity and glutathione (GSH) levels were noted [15].

Under basal conditions, the cytosolic protein Keap1 facilitates the continuous ubiquitination and consequent degradation of nuclear factor erythroid 2‐related factor 2 (Nrf2). However, upon oxidative stress, the autophagic adapter p62 interacts with Keap1, disrupting the Keap1‐Nrf2 complex. This interaction liberates Nrf2, allowing its nuclear translocation and subsequent induction of genes associated with antioxidant defense and detoxification [16]. Nrf2 acts as the main transcriptional controller of cellular antioxidant defenses, coordinating the expression of protective genes like heme oxygenase‐1 (HO‐1) and GPX4 [17]. Substantial evidence demonstrates Nrf2's anti‐ferroptotic activity across multiple cell lineages through amelioration of oxidative stress, particularly in cardiovascular pathologies [18]. In the context of DIC, accumulating research has established Nrf2 as a critical modulator of ferroptosis resistance.

The PI3K/AKT signaling pathway serves as a central regulator of multiple physiological processes by activating downstream effectors involved in cell cycle progression and proliferation [19]. PI3K, a lipid kinase, phosphorylates the D3 hydroxyl group of inositol rings within phosphoinositide lipids upon activation by various extracellular signals [20]. Once activated, PI3K recruits downstream signaling proteins such as AKT, a serine/threonine kinase that exists primarily in three subtypes: AKT1, AKT2, and AKT3. Among these, AKT1 exhibits the broadest tissue distribution [21]. Activation of AKT further propagates the signal to downstream pathways, influencing essential cellular functions [22]. Recent investigations have increasingly focused on the PI3K/AKT/Nrf2 pathway due to its crucial involvement in oxidative stress defense [23]. This signaling cascade mediates cellular survival in various tissues, particularly in cardiomyocytes [24]. Accumulating evidence supports the therapeutic value of targeting the PI3K/AKT pathway in DIC, highlighting the PI3K/AKT/Nrf2 axis as a promising molecular focus for intervention [25].

Morroniside (Mor) is an active compound present in Cornus officinalis, a plant utilized as both food and traditional Chinese medicine in Japan, Korea, and China for more than 2000 years [26]. Mor exhibits a polypharmacological profile. Its effects span reducing oxidative stress, inhibiting carcinogenesis, and attenuating inflammation in previous studies [27]. Experimental investigations have demonstrated that Mor enhances the activity of both GSH and SOD in biological tissues [28]. Furthermore, the activation of the Keap1/Nrf2 signaling pathway by Mor, along with the inhibition of malondialdehyde (MDA) formation, helps to mitigate oxidative stress, with MDA being a crucial lipid peroxidation product. Although the antioxidant and anti‐ferroptotic activities of Mor have been reported in other disease contexts, its role in DIC and the upstream signaling mechanism that links Mor to Nrf2 activation remain unclear [29]. Therefore, this study aimed to determine whether Mor attenuates DIC and to clarify whether Mor suppresses ferroptosis and oxidative stress through the PI3K/AKT/Nrf2/HO‐1 pathway. To our knowledge, this is the first study to identify the PI3K/AKT/Nrf2/HO‐1‐mediated anti‐ferroptotic axis as a key mechanism underlying Mor‐mediated cardioprotection against DIC.

## 2 Methods and Materials

### 2.1 Reagents

Mor (#HY‐N0532), RSL3 (#HY‐100218A), and LY294002 (#HY‐10108) were purchased from MedChemExpress, while Dox (#D1515) and Ferrostatin‐1 (Fer‐1, #SML0583) were sourced from Sigma‐Aldrich. The above reagents were used for animal model construction and inhibition of ferroptosis and PI3K activity. The following primary antibodies were used in this study: PTGS2 (#12282, CST), GPX4 (#A11243, ABclonal), PI3K (#AF6241, Affinity), p‐PI3K (#AF3241, Affinity), Nrf2 (#12721, CST), Histone‐ H3 (#A2348, ABclonal), AKT (#10176‐2‐AP, Proteintech), p‐AKT (#66444‐1‐Ig, Proteintech), HO‐1 (#10701‐1‐AP, Proteintech), Cle‐Caspase‐3 (#AF7022, Affinity), GAPDH (#10494‐1‐AP, Proteintech). The above antibodies were used for immunofluorescence assays and western blot. The detailed sources and catalog numbers of all reagents were provided in Table 1 of the supporting materials.

### 2.2 Animals

All animal procedures were conducted in accordance with the guidelines established by the American Veterinary Medical Association and adhered to the ARRIVE guidelines. The study was reviewed and approved by the Institutional Animal Care and Use Committee of Wenzhou Medical University (wydw2025‐0235).

For the experiments, 6‐ to 8‐week‐old C57BL/6 mice were obtained from Zhejiang Vital River Laboratory Animal Technology Co. A noticeable decline in movement and reduced self‐grooming behavior was adopted as a surrogate marker for end‐stage diseases. At the conclusion of the experiment, the mice were anesthetized with isoflurane (5% for initial induction, followed by 2% for sustained anesthesia) (RWD, Shenzhen, China). Euthanasia was then performed via cervical dislocation. Following this, tissue and organ samples were harvested, and blood samples were obtained from the abdominal aorta for further analysis. All procedures were strictly followed the ARRIVE guidelines for ethical animal research.

### 2.3 Experimental Design

[1] Control (Ctrl) group: Twenty mice were divided into four cages, and mice received a saline vehicle only via intragastric (ig) administration [2]. DIC group: Subjects were maintained on intragastric saline for 5 weeks, with additional weekly intraperitoneal (ip) Dox injections (5 mg/kg) initiated after the first week and continued for 4 weeks [3]. Mor+DIC group: Following 1 week of Mor pretreatment (5 mg/kg/day, ig), mice received concurrent weekly Dox (5 mg/kg, ip) for 4 weeks while maintaining daily Mor administration (total 5 weeks).

In vitro studies: The DIC model was established using 1 μM Dox, with 10 μM Mor employed as a therapeutic intervention. PI3K inhibition was achieved with 10 μM LY294002, and ferroptosis inducer was achieved with 1 μM RSL3. H9c2 cells were incubated with Dox, LY294002, or RSL3 for 24 h. For protection experiments, cells were pretreated with Mor for 24 h before Dox administration, leading to a cumulative Mor exposure time of 48 h.

### 2.4 Echocardiography

An ultrasound system was utilized to conduct echocardiography in order to evaluate the impact of Mor on cardiac function. Prior to imaging, the mice were placed on a temperature‐controlled platform, deeply anesthetized, and their thoracic fur was removed. A trained technician, blinded to the treatment groups, performed the measurements and calculated left ventricular fractional shortening (LVFS) and left ventricular ejection fraction (LVEF) by averaging values obtained from a minimum of six consecutive cardiac cycles.

### 2.5 Cell Culture

The H9c2 and AC16 cell lines were cultured in DMEM (Gibco) enriched with 10% heat‐inactivated FBS (Gibco) under standard conditions (37°C, 5% CO_2_, humidified atmosphere). Cells were obtained directly from the American Type Culture Collection and used between passages 5–15.

### 2.6 Cell Viability

H9c2 cardiomyocytes were seeded in 96‐well culture plates at appropriate densities. A volume of 100 μL containing 2000 cells was added to each well. After experimental treatment, 10 μL of CCK‐8 working solution was added, followed by 1 h of co‐incubation. The absorbance was then measured at 450 nm. Cell viability was measured using the Cell Counting Kit‐8 (CCK‐8) (#C0038, Beyotime), following the instructions provided by the manufacturer.

### 2.7 Biochemical Analysis

After completing the experimental treatments, we collected both culture supernatants and mouse serum samples following the protocols provided with each assay kit. Protein extracts were prepared from either cardiac tissue or cultured cells. Subsequent biochemical analyses were performed using commercial kits to measure: SOD (#A001‐2‐2), lactate dehydrogenase (LDH) (#A020‐2‐2), GSH (#A006‐2‐1), catalase (CAT) (#A007‐2‐1), MDA (#A003‐2‐1), and creatine kinase‐MB isoenzyme (CK‐MB) (#E006‐1‐1) were performed with assay kits from Nanjiang Jiancheng. The concentration of cardiac troponin T (cTnT) in serum was determined through an enzyme‐linked immunosorbent assay (ELISA) (#E‐EL‐M1801, Elabscience).

### 2.8 Hematoxylin and Eosin (H&E) Staining

Cardiac specimens were fixed with 4% paraformaldehyde, followed by dehydration through a graded ethanol series, clearing in xylene, and embedding in paraffin. Using a microtome, 6‐μm serial sections were obtained from the paraffin blocks. After deparaffinization, the sections were stained following the standard H&E protocol. Finally, the stained sections were observed and captured under a light microscope.

### 2.9 ROS or DHE Staining Assay

H9c2 cardiomyocytes were plated in 6‐well culture plates at a density of 10,000 cells per well in 6 mL of culture medium. After experimental treatment, the cells were treated with the 3 μL DHE (#S0063, Beyotime)/ROS (#S0033S, Beyotime) solution as directed by the manufacturer. Following a 35‐min staining period at 37°C, the solution was aspirated, and cells received four PBS washes. Fluorescence was stabilized using an anti‐quenching medium.

### 2.10 Cellular Immunofluorescence

At room temperature, H9c2 cardiomyocytes were fixed in 4% paraformaldehyde for a duration of 20 min. Using 0.25% Triton, cell membranes were permeabilized for 9 min, followed by a 3‐h block with 5% BSA. The primary antibody was incubated at 4°C overnight. The next day, cells were treated with appropriate secondary antibodies for 1.5 h at room temperature, followed by DAPI staining for the nuclei.

### 2.11 Iron Content Detection

Intracellular and myocardial iron concentrations were quantified in H9c2 cell lysates and cardiac tissue homogenates respectively, following the guidelines from the manufacturer, a commercial Iron Assay Kit (#ab83366, Abcam) was used.

### 2.12 Transient Transfection of siRNA

Nrf2 small interference RNA (siRNA) sense sequences were 5′‐ GCCTTACTCTCCCAGTGAATA‐3′. The Nrf2‐siRNA sequence was subjected to NCBI BLASTn screening against the rat transcriptome/genome database, and no significant off‐target transcripts were identified. SiRNA transfection was performed in H9c2 cells following the manufacturer's protocol for Lipofectamine 3000 (Invitrogen).

### 2.13 Separation of Nuclear and Cytoplasmic Proteins

Cells were washed with PBS and gently detached with a cell scraper. Proceed with subsequent steps according to the kit manufacturer's instructions (#P0027, Beyotime). After centrifugation, the supernatant was gently aspirated. The Cell pellet was resuspended in cytoplasmic extraction reagent A containing PMSF, vigorously vortexed, and incubated on ice for 10 min. Following the addition of cytoplasmic extraction reagent B, the sample was vortexed again and centrifuged at 4°C. The resulting supernatant was collected. The remaining pellet was resuspended in nuclear extraction reagent with PMSF and subjected to intermittent vigorous vortexing every 3 min for 30 min on ice. After final centrifugation, the nuclear extract was aliquoted into pre‐chilled tubes for immediate use or storage at –80°C.

### 2.14 Western Blotting Analysis

With RIPA lysis buffer, cellular and tissue protein extracts were prepared, and protein concentrations were determined through a BCA assay (#P0013B, Beyotime). Protein samples were separated based on molecular weight using SDS‐PAGE and subsequently transferred onto PVDF membranes (#ISEQ. 85 R, Millipore Corporation). Membranes underwent a blocking step for 2.5 h with 5% skim milk at 4°C before being exposed to primary antibodies overnight. Next day, they were incubated with a goat anti‐rabbit IgG secondary antibody (#bl003a, Biosharp) for 3 h at room temperature. Western blot results were captured with a Bio‐Rad ChemiDoc XRS+ system (Bio‐Rad) and processed through Image Lab analysis software. Relative protein expression was determined by densitometric quantification in ImageJ after background subtraction and reference normalization.

### 2.15 Pharmacological Network Analysis

The putative molecular targets of Mor were determined by an integrated computational strategy employing three prediction platforms: SwissTargetPrediction (http://www.swisstargetprediction.ch/), SuperPred (https://prediction.charite.de/), and. Disease‐associated genes related to DIC were retrieved from GeneCards (https://www.genecards.org/) and OMIM (https://www.omim.org/) databases. By intersecting these two datasets, we identified potential therapeutic targets through which Mor might exert its cardioprotective effects against DIC. Protein‐protein interaction networks were generated via the STRING database (https://cn.string-db.org/) and subsequently visualized and analyzed using Cytoscape.

For functional characterization, the identified targets were subjected to comprehensive bioinformatics analyses. The analyses for Gene Ontology (GO) enrichment and KEGG pathways were carried out in RStudio, with *p* < 0.05 as the criterion for statistical significance.

### 2.16 Molecular Docking Followed by Dynamics Simulations

The ligand structure was downloaded from PubChem in SDF format and subjected to energy minimization with ChemBio3D Ultra 14.0. The PI3K protein structure was obtained from the Protein Data Bank and prepared in PyMOL 2.3.0 by removing water molecules, ions, and nonessential ligands. Subsequently, both the protein and ligand were processed in AutoDockTools 1.5.6 for hydrogen addition, Gasteiger charge assignment, and pdbqt file generation. The binding pocket was predicted using POCASA 1.1. Molecular docking was performed using AutoDock Vina 1.1.2. The docking grid box was centered at *x* = 3.156, *y* = 4.259, and *z* = 20.274, with dimensions of 60 Å × 60 Å × 60 Å, covering the predicted PI3K binding pocket.

All‐atom molecular dynamics simulations were conducted on the docked protein‐ligand complex using GROMACS 2022.4. The protein was parameterized with the Amber14SB force field, and ligand topologies were generated using ACPYPE and Antechamber. The system was solvated in a cubic box with a 1 nm boundary distance from the complex, using the TIP3P water model and neutralized with sodium and chloride ions. Energy minimization was carried out using the steepest descent method, followed by equilibration under NVT and NPT ensembles at 300 K and 101.325 kPa, respectively. Production simulations were run for 100 ns, generating 10,000 trajectory frames. Trajectory analysis included RMSD, RMSF, Rg, and SASA. Binding free energy was calculated with gmx_mmpbsa using 100 frames from the equilibrated trajectory.

### 2.17 Statistical Analysis

Data analysis utilized SPSS 22.0. Parametric data appear as mean ± SD. Between‐group differences were evaluated by: (1) Student's *t*‐test (two groups), or (2) ANOVA + Duncan's T3 (multiple groups), with *p* < 0.05 considered significant.

## 3 Results

### 3.1 Analysis of Mor and DIC Using Bioinformatics

**Figure 1:**
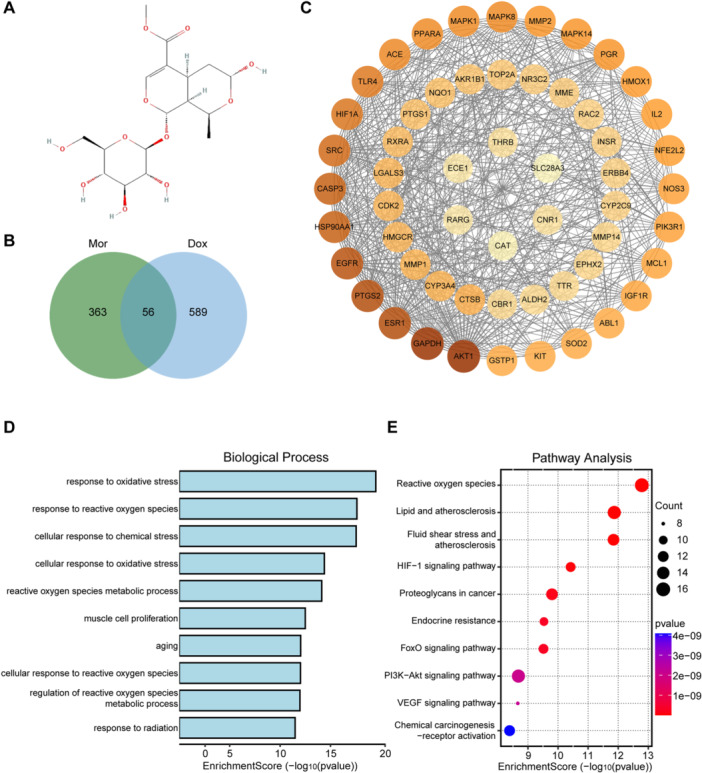
Bioinformatics integration between Mor and DIC. (A) The chemical structure of Mor. (B) The common targets found in both the therapy (Mor) and the disease (DIC). (C) Identified targets and their protein interaction network. (D) Analysis of GO enrichment using genes with differential expression. (E) Using differentially expressed genes for KEGG pathway analysis.

Mor (C_17_H_26_O_11_), an iridoid glycoside compound, is chiefly found in Cornus and elderberry and is noted for its potent pharmacological actions, such as opposing ischemia and hypoxia, alleviating oxidative stress, and inhibiting ferroptosis. Using Pubchem, the chemical structure of Mor was depicted (Figure 1A). 419 targets of Mor were obtained through SuperPred, PharmMapper, and SwissTargetPrediction, and 645 targets of DIC were obtained through GeneCards and OMIM. There are 56 overlapping genes between the two, which may be the targets of Mor for the treatment of DIC (Figure 1B). The PPI analysis revealed crucial targets, which may play an important role in DIC treatment using Mor (Figure 1C). GO enrichment analysis results indicate that Mor is involved in multiple biological processes (BP) related to DIC, including the response to oxidative stress and reactive oxygen species (Figure 1D). According to KEGG analysis, the reactive oxygen species pathway and PI3K‐AKT signaling pathway ranked among the top 10 pathways (Figure 1E).

### 3.2 In Vitro, Mor Inhibited Ferroptosis and Oxidative Stress in DIC

**Figure 2:**
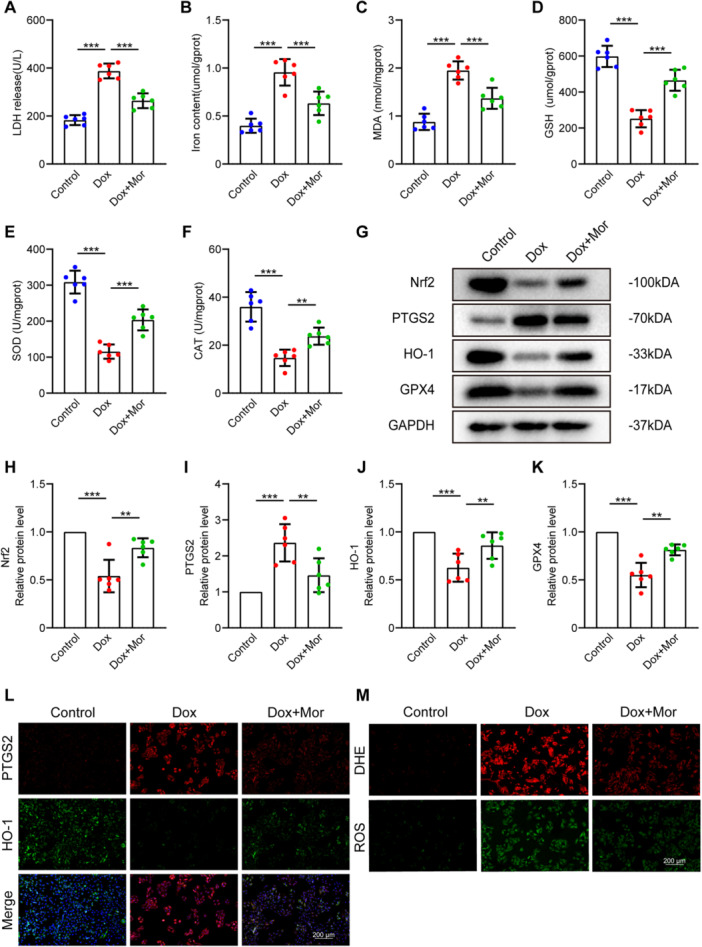
In vitro experiments showed that Mor diminished the ferroptosis and oxidative stress resulting from Dox. (A) Cytotoxicity was evaluated via LDH assay in all experimental groups, *n* = 6. (B–F) Iron content, MDA, GSH, SOD, and CAT served as markers of ferroptosis and oxidative stress in all experimental groups, *n* = 6. (G) Western blotting was employed across all groups to determine the levels of ferroptosis and oxidative stress. (H–K) The levels of Nrf2, PTGS2, HO‐1, and GPX4 were assessed following western blot analysis, *n* = 6. (L) Immunofluorescence staining was used to detect PTGS2 and HO‐1 in all experimental groups, 100x, *n* = 3. (M) In the context of oxidative stress staining, DHE is marked by red and ROS by green, 100x, *n* = 3. Data are means ± SD, *p < 0.05, **p < 0.01, ***p < 0.001.

To figure out the best concentration of Mor for DIC, we initially employed the CCK‐8 assay to measure cell viability. Through this measurement, we identified that 10 μM of Mor was the most suitable concentration, and this concentration was applied in the following experiments (Figure S1A). Once the DIC model was established, observations revealed that Mor reduced cytotoxicity (Figure 2A). Notably, independent experiments demonstrated that Mor alone had no impact on either cell viability or cytotoxicity levels. (Figure S1B,C). In the pharmacological network analysis, GO enrichment and KEGG pathway analysis indicated that the protective effect of Mor might be associated with the modulation of oxidative stress and ROS. Oxidative stress and the generation of ROS are closely intertwined with ferroptosis [30]. It has been reported that Nrf2 and its downstream gene expression are closely associated with oxidative stress and ferroptosis [31].

Additionally, we assessed markers of ferroptosis and oxidative stress. Our results showed that Mor notably decreased iron content and MDA levels, while enhancing GSH, SOD, and CAT levels (Figure 2B–F). HO‐1 is an antioxidant gene activated by Nrf2. As for ferroptosis, PTGS2 and GPX4 serve as its characteristic molecular markers. Through Western blotting, PTGS2 expression exhibited a significant upregulation in DIC, whereas the expression levels of Nrf2, HO‐1, and GPX4 showed a downward trend (Figure 2G–K). On the contrary, Mor treatment led to a reversal of these alterations. The outcomes of the cell fluorescence experiments are in line with those of previous studies (Figures 2L and S1D,E). Additionally, DHE/ROS staining revealed that Mor suppressed ROS generation (Figures 2M and S1F,G). To investigate whether Mor regulates the nuclear translocation of Nrf2, we measured Nrf2 expression in the nuclear fraction by Western blot. The results showed that Dox inhibited the nuclear import of Nrf2, whereas Mor treatment facilitated it (Figure S1H,I). This finding was further corroborated by immunofluorescence staining of Nrf2 (Figure S1J). Collectively, these data suggest that Mor can effectively attenuate ferroptosis and oxidative stress in DIC.

### 3.3 In Vitro, Mor Attenuated DIC by Inhibiting Ferroptosis

**Figure 3:**
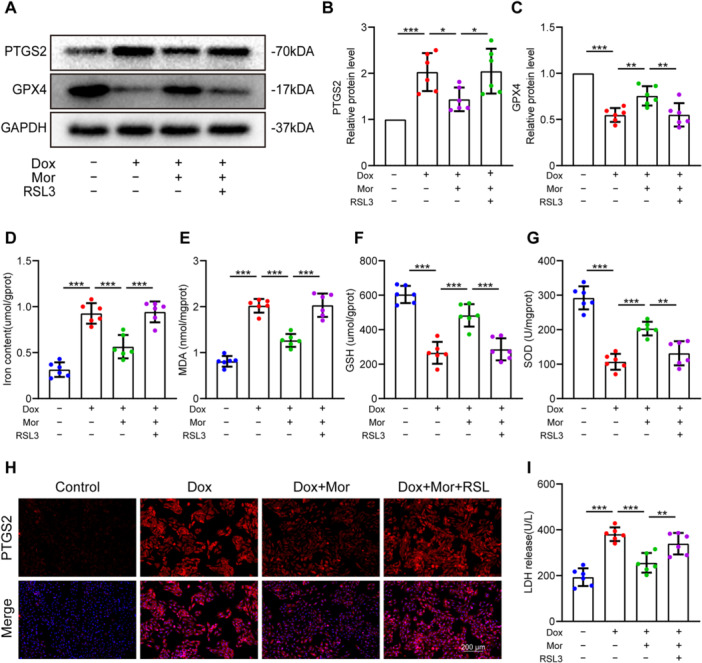
In vitro, the ferroptosis inducer significantly weakened the protective effect of Mor in DIC. (A) Western blotting was employed across all groups to determine the levels of ferroptosis. (B, C) The levels of PTGS2 and GPX4 were assessed following western blot analysis, *n* = 6. (D–G) Iron content, MDA, GSH, and SOD served as markers of ferroptosis and oxidative stress in all experimental groups, *n* = 6. (H) Immunofluorescence staining was used to detect PTGS2 in all experimental groups, 100x, *n* = 3. (I) Cytotoxicity was evaluated via LDH assay in all experimental groups, *n* = 6. Data are means ± SD, *p < 0.05, **p < 0.01, ***p < 0.001.

To further explore whether ferroptosis is involved in Mor's potential role in DIC, we used the GPX4‐specific inhibitor RSL3, known as a ferroptosis inducer. As anticipated, RSL3 blocked Mor's beneficial impacts on ferroptosis‐related parameters such as iron content, MDA, GSH, SOD, PTGS2, and GPX4 (Figure 3A–G). Cellular fluorescence also partially corroborated this result (Figures 3H and S1K). Moreover, administering RSL3 eliminated Mor's protective effects against DIC (Figures 3I and S1L).

### 3.4 In Vitro, Mor Attenuated Ferroptosis, Oxidative Stress and DIC Depend on Activating Nrf2

**Figure 4:**
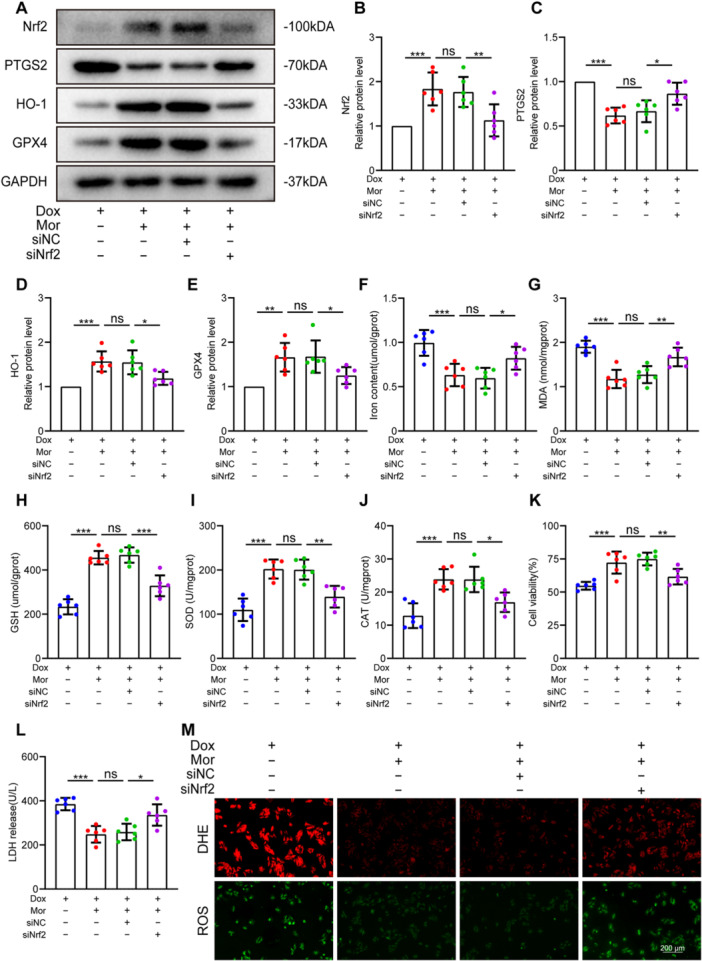
In vitro, the inhibition of ferroptosis, oxidative stress, and DIC by Mor depends on the activation of Nrf2. (A) Western blotting was employed across all groups to determine the levels of ferroptosis and oxidative stress. (B–E) The levels of Nrf2, PTGS2, HO‐1, and GPX4 were assessed following western blot analysis, *n* = 6. (F–J) Iron content, MDA, GSH, SOD, and CAT served as markers of ferroptosis and oxidative stress in all experimental groups, *n* = 6. (K, L) Cytotoxicity was evaluated via cell viability and LDH assay in all experimental groups, *n* = 6. (M) In the context of oxidative stress staining, DHE is marked by red and ROS by green, 100x, *n* = 3. Data are means ± SD, *p < 0.05, **p < 0.01, ***p < 0.001.

To explore the interaction between Nrf2 and ferroptosis in DIC within an in vitro system, H9c2 cells were transfected with Nrf2 small interfering RNA (Nrf2‐siRNA). Through western blot analysis, we observed that Nrf2‐siRNA significantly reduced Nrf2 protein expression (Figure 4A,B). Notably, Nrf2‐siRNA attenuated Mor's protective effects against Dox‐induced ferroptosis and oxidative stress. Specifically, Mor's inhibitory effect on PTGS2 protein expression was abrogated, while its promoting effects on HO‐1 and GPX4 were suppressed (Figure 4A,C–E). Nrf2‐siRNA also reversed Mor's impacts on iron content, MDA, GSH, SOD, and CAT levels (Figure 4F–J). DHE/ROS staining experiments further corroborated the findings (Figures 4M and S1M,N). In the context of DIC, Nrf2‐siRNA reversed Mor's protective effects in H9c2 cells, including the reduction in LDH release and the enhancement of cell viability (Figure 4K,L). Collectively, these results suggest that Mor's protective mechanisms are contingent upon activating Nrf2.

### 3.5 In Vitro, Mor Activated the PI3K/AKT Signaling Pathway in DIC, While LY294002 Nullifies the Activating Effect

**Figure 5:**
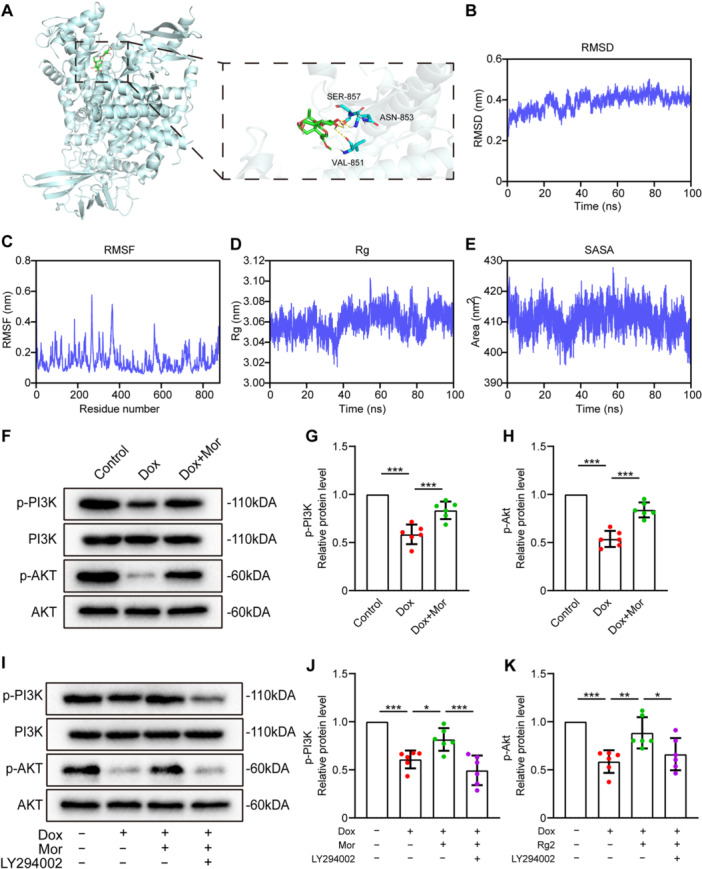
In vitro studies showed that Mor triggered the PI3K/AKT signaling pathway in DIC, while LY294002 inhibited this activation. (A) The 3D‐molecular docking of PI3K (Blue) and Raf‐1 (Green). The residues involved in hydrogen bonding are visible in a zoomed‐in view of the binding pocket. (B–E) RMSD, RMSF, Rg, and SASA values of the protein‐ligand complexes over time. (F) Western blotting was employed across all groups to determine the levels of PI3K and AKT. (G, H) The levels of PI3K and AKT were assessed following western blot analysis, *n* = 6. (I) Western blotting was employed across all groups to determine the levels of PI3K and AKT. (J, K) The levels of PI3K and AKT were assessed following western blot analysis, *n* = 6. Data are means ± SD, *p < 0.05, **p < 0.01, ***p < 0.001.

KEGG analysis suggested that Mor may be associated with the PI3K/AKT signaling pathway in the context of DIC. Dox could cause cardiotoxicity by inhibiting the activity of PI3K/AKT. Molecular docking was conducted to examine the binding interaction between Mor and specific binding sites on the PI3K protein. The binding energy of the PI3K–Mor complex was computed to be –7.4 kcal/mol, with hydrogen bonds formed between the ligand and residues SER854, ASN853, and VAL851 of PI3K (Figure 5A). To further validate the binding, molecular dynamics simulations of the PI3K–Mor system were conducted. The root mean square deviation (RMSD) values fluctuated mainly between 0.2 and 0.4 nm and reached a relatively stable plateau after approximately 50 ns, indicating that the system achieved conformational equilibrium during the latter half of the simulation. Root mean square fluctuation (RMSF) analysis revealed that most residues exhibited fluctuations below 0.4 nm, suggesting minimal perturbation of the protein backbone upon ligand binding. The radius of gyration (Rg) of PI3K remained around 3.06 nm with minor variations (3.01–3.10 nm) and became stable after approximately 50 ns, reflecting sustained structural compactness throughout the equilibrated phase. Similarly, the solvent‐accessible surface area (SASA) values ranged between 395 and 428 nm^2^ with only slight fluctuations, demonstrating overall structural stability of the complex. Therefore, the subsequent binding free‐energy calculation was performed using frames extracted from the equilibrated trajectory (Figure 5B–E).

We found that Mor increased PI3K/AKT activity that was originally inhibited by Dox (Figure 5F–H). While we demonstrated that Mor could counteract Dox‐mediated inhibition of PI3K and AKT phosphorylation, the relationship between this effect and the alleviation of ferroptosis and oxidative stress remains unclear. To further investigate, we utilized LY294002, a PI3K inhibitor, to suppress PI3K and AKT phosphorylation, aiming to observe the impact of Mor on ferroptosis and oxidative stress under these conditions (Figure 5I–K).

### 3.6 In Vitro, Mor Attenuated Ferroptosis, Oxidative Stress and DIC by Activation of PI3K/AKT/Nrf2/HO‐1 Pathway

**Figure 6:**
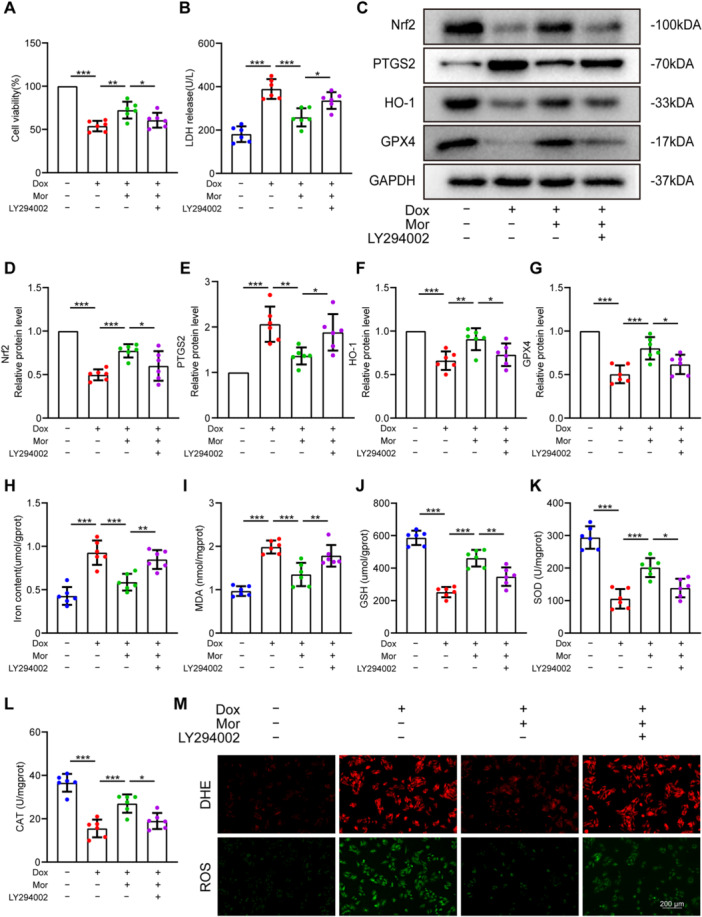
In vitro, Mor activates Nrf2/HO‐1 to alleviate ferroptosis, oxidative stress, and DIC, which depend on the activation of the PI3K/AKT pathway. (A, B) Cytotoxicity was evaluated via cell viability and LDH assay in all experimental groups, *n* = 6. (C) Western blotting was employed across all groups to determine the levels of ferroptosis and oxidative stress. (D–G) The levels of Nrf2, PTGS2, HO‐1, and GPX4 were assessed following western blot analysis, *n* = 6. (H–L) Iron content, MDA, GSH, SOD, and CAT served as markers of ferroptosis and oxidative stress in all experimental groups, *n* = 6. (M) In the context of oxidative stress staining, DHE is marked by red and ROS by green, 100x, *n* = 3. Data are means ± SD, *p < 0.05, **p < 0.01, ***p < 0.001.

Nrf2 activation, which was regulated by the PI3K/AKT pathway, had been demonstrated to modulate oxidative stress levels [32]. To further investigate the role of the PI3K/AKT signaling pathway on ferroptosis and oxidative stress, we used LY294002 to investigate the subsequent process. It was obvious‌ that LY294002 removed the therapeutic impact of Mor, lowered cell survival, and raised cytotoxicity (Figure 6A,B). The detection of ferroptosis of H9c2 cells in all groups indicated that ferroptosis‐related proteins in the LY294002 group were increased compared with the Mor group. The inhibition of oxidative stress by Mor was also reversed by LY294002 (Figure 6C–G). Meanwhile, low levels of p‐PI3K led to an increase in iron content, MDA accumulation, and a decrease in the production of CAT, SOD, and GSH (Figure 6H–L). DHE/ROS staining also demonstrated that the reduction of oxidative stress levels induced by Mor was reversed by LY294002 (Figures 6M and S1O,P).

### 3.7 In Vivo, Mor Attenuated DIC, Ferroptosis and Oxidative Stress

**Figure 7:**
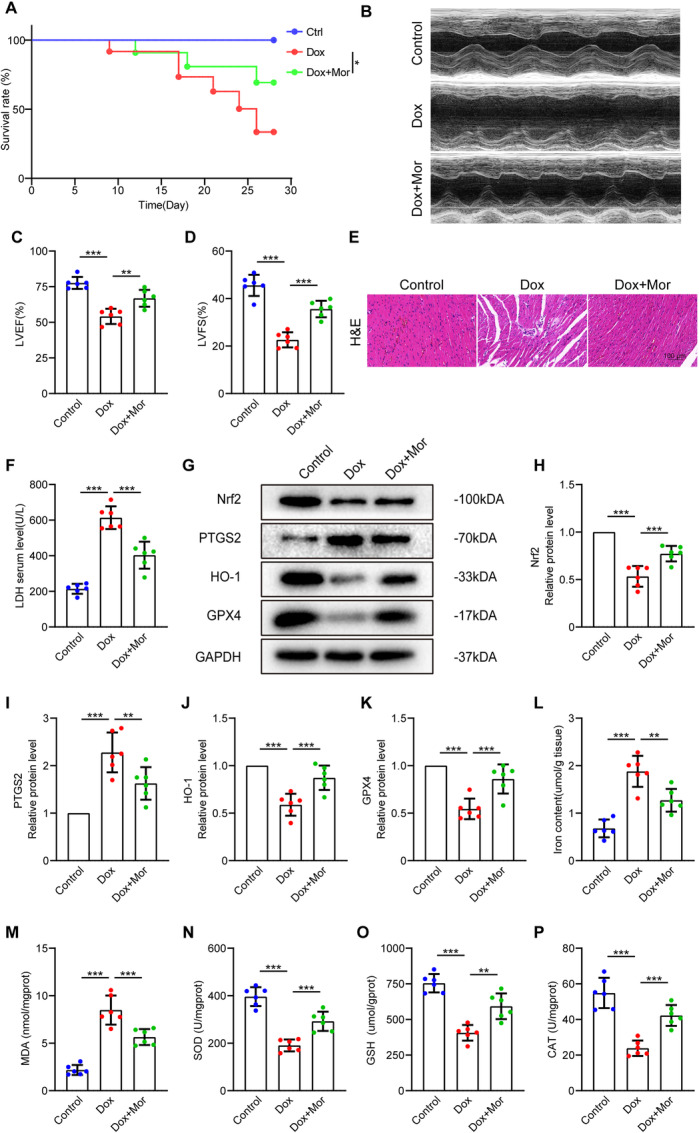
In the DIC model in vivo, Mor also had a protective effect. (A) Measurements of survival rates were taken for every group. (B) Representative echocardiographic images from each experimental group, *n* = 6. (C, D) LVEF and LVFS, *n* = 6. (E) Representative images of H&E staining, 200x, *n* = 3. (F) The concentration of LDH in mouse serum, *n* = 6. (G) Western blotting was employed across all groups to determine the levels of ferroptosis and oxidative stress. (H–K) The levels of Nrf2, PTGS2, HO‐1, and GPX4 were assessed following western blot analysis, *n* = 6. (L–P) Iron content, MDA, GSH, SOD, and CAT served as markers of ferroptosis and oxidative stress in all experimental groups, *n* = 6. Data are means ± SD, *p < 0.05, **p < 0.01, ***p < 0.001.

We established a DIC model to study Mor's role in DIC, with the goal of gaining a deeper understanding of Mor's function in vivo (Figure S1Q). Based on CK‐MB and cTnT detection, the optimal dose of Mor was determined to be 5 mg/kg/d (Figure S2A,B). We found that the concentration of Mor by itself had no effect on mouse myocardium (Figure S2C–E). As previous studies have shown, DIC mice experienced increased mortality. The administration of Mor significantly boosted their survival rate (Figure 7A). In vivo echocardiographic assessment revealed key metrics of left ventricular performance (Figure 7B). A marked decrease in both LVEF and LVFS was observed, reflecting substantial myocardial injury in the DIC mice (Figure 7C,D). Administration of Mor, however, was found to attenuate the impaired cardiac function induced by Dox. Morphological assessment revealed that Dox treatment led to noticeable inflammatory infiltration and alterations in tissue architecture, whereas administration of Mor markedly attenuated these pathological changes (Figure 7E). According to biochemical analysis, Mor markedly reduced the levels of the myocardial injury marker LDH (Figure 7F). Mice in the DIC model exhibited restricted growth. Correspondingly, a reduction in the heart‐to‐tibia index was observed in the DIC group (Figure S2F). However, Mor effectively reversed these effects.

Western blot analysis of ferroptosis‐ and oxidative stress‐associated proteins revealed that Mor reduced the extent of ferroptosis and oxidative stress in vivo (Figure 7G–K). Further detection of the levels of iron content, MDA, GSH, SOD, and CAT could also verify this result (Figure 7L–P).

## 4 Discussion

Collectively, these findings established novel therapeutic potential for DIC: The findings suggested that Mor might attenuate ferroptosis, inhibit oxidative stress, and ultimately safeguard cardiomyocytes by activating the PI3K/AKT/Nrf2/HO‐1 pathway. The use of Mor in DIC mice showed an increase in survival rates and a reduction in myocardial injury markers, offering a promising new drug and target for DIC therapy.

Dox, a commonly used anthracycline in clinical oncology, faces limitations because of its pronounced cardiotoxicity [33]. Although DIC mechanisms are complex, it is generally accepted that cell death processes like ferroptosis, apoptosis, pyroptosis, and autophagy play a major role [34]. Furthermore, disturbances in iron metabolism and overactivation of ROS are key factors in DIC [12, 35]. Our study was centered on the role of Mor in ferroptosis and oxidative stress triggered by Dox. Ferroptosis requires three key elements: reactive iron species, oxidizable polyunsaturated fatty acid (PUFA) in membranes, and insufficient GPX4/GSH antioxidant defense. The convergence of these factors leads to lethal lipid peroxidation [36]. Evidence suggests that targeting ferroptosis rather than other forms of programmed cell death leads to greater survival benefits in Dox‐treated mouse models [12]. Some research suggests that inhibiting ferroptosis might significantly attenuate DIC and protect cardiomyocytes. By inhibiting mitochondrial ROS and ferroptosis, WGX50 alleviates the cardiotoxic effects of Dox [37]. Through the adjustment of a ferroptosis‐dependent pathway, salidroside mitigates cardiomyopathy caused by Dox [38].

Nrf2 is essential for preserving cellular redox homeostasis through the coordinated induction of cytoprotective genes [39]. When oxidative stress occurs, Nrf2 enters the nucleus and connects with the antioxidant response element (ARE), enhancing the production of detoxification enzymes and antioxidant proteins [40]. Activation of Nrf2 is critical for defending cells against oxidative damage and enhancing their viability. Additionally, improper regulation of Nrf2 activity has been linked to the development of several diseases, such as cancer and metabolic disorders [41]. Our investigation demonstrates that Mor upregulates both Nrf2 and its downstream antioxidant targets, thereby suppressing ferroptosis. This protective effect was significantly diminished upon Nrf2 silencing. Multiple independent studies have documented the protective role of Nrf2 activation against ferroptosis in DIC models. Calycosin alleviates ferroptosis and reduces heart damage caused by Dox via the Nrf2/SLC7A11/GPX4 signaling pathway [42]. Through HuR‐dependent m6A regulation, FTO mitigates DIC by blocking ferroptosis via P53‐P21/Nrf2 activation [43].

Cell death is significantly influenced by the PI3K/AKT signaling pathway [44]. Studies have found that AKT inactivation correlates with DIC, while higher levels of AKT phosphorylation help cardiomyocytes survive and decrease DIC [45, 46]. Activating the pathway supports cell survival, maintains redox balance, and protects against oxidative stress, with research indicating that the PI3K/AKT pathway can enhance the Nrf2/HO‐1 axis to protect cells [32, 47]. Although our experiments focused on PI3K/AKT‐mediated Nrf2 activation, Mor‐mediated cardioprotection may also involve crosstalk with other transcriptional regulators. PPAR signaling is closely associated with myocardial lipid metabolism, mitochondrial function, and ferroptosis susceptibility. Recent evidence in DIC models suggests that regulation of the PPAR pathway can reduce lipid accumulation and ferroptosis‐related injury [48]. Given that ferroptosis depends on the availability of oxidizable membrane lipids, PPARγ‐related lipid metabolic remodeling may cooperate with the Nrf2/GPX4 antioxidant axis to determine the ferroptotic threshold in cardiomyocytes. In addition, p53 has been implicated in DIC‐related ferroptosis through the regulation of iron homeostasis and antioxidant defense. For example, p53 signaling can influence SLC7A11/GPX4‐mediated ferroptosis resistance, whereas modulation of p53 degradation has been shown to alleviate DIC ferroptosis and cardiac dysfunction [43, 49]. Therefore, the PI3K/AKT/Nrf2/HO‐1 pathway identified in the present study may represent a central protective axis, while PPARγ‐ and p53‐associated signaling may act as complementary regulatory nodes. Further work is needed to determine whether Mor directly modulates these transcriptional networks in DIC.

Numerous studies have discovered that Mor benefits the cardiovascular system. Mor exhibits cardioprotective properties following myocardial infarction, preserving cardiomyocyte viability while actively stimulating cardiac repair mechanisms [50, 51]. Wen et al reported that Mor inhibits apoptosis in cardiomyocytes caused by high glucose levels [52]. Compared with other natural products investigated for DIC, the effective protective dose of Mor in our study was relatively low. In the present in vivo experiments, Mor was administered at 2.5 and 5 mg/kg/day, and 5 mg/kg/day showed clear cardioprotective efficacy. By contrast, previous studies have reported quercetin at oral doses of 10–50 mg/kg in Dox‐induced cardiac injury models, with cardioprotection associated with Nrf2/HO‐1 activation [53]. Resveratrol has also been shown to protect against DIC by attenuating ferroptosis through MAPK pathway modulation, and resveratrol doses in DIC‐related animal studies are commonly higher than the Mor dose used here [54]. Although differences in animal species, Dox regimens, administration routes, and pharmacokinetic properties preclude a direct potency comparison, these findings suggest that Mor may exert meaningful cardioprotective activity at a comparatively modest dose. This dose‐related advantage, together with its multi‐target antioxidant and anti‐ferroptotic profile, supports the potential translational value of Mor as an adjunctive strategy for DIC.

Our study had several limitations. Firstly, we did not evaluate other pathways potentially involved in DIC. Processes such as apoptosis, necrosis, and autophagy are also known to contribute to DIC. Secondly, the use of specific activators and inhibitors targeting the Nrf2/HO‐1 pathway would help further validate the regulatory role of Mor in this mechanism. Thirdly, mass spectrometry‐based profiling of serum absorption components, along with in vivo transcriptomic analysis of cardiac tissue, would be important to elucidate the downstream targets and pathways modulated by Mor. Fourthly, the present study mainly used in vitro pharmacological blockade and Nrf2‐siRNA to verify pathway dependence. In vivo blocking experiments were not performed, which limits the strength of causal evidence at the animal level. Future studies should employ Nrf2 knockout mice, cardiomyocyte‐specific Nrf2 conditional knockout models, or adeno‐associated virus (AAV)‐mediated cardiac‐specific Nrf2 knockdown/overexpression to validate the requirement of Nrf2 activation for Mor‐mediated cardioprotection in vivo. Finally, we further validated the cardioprotective effect of Mor using human‐derived AC16 cardiomyocytes (Figure S2G–I). Our findings revealed that Mor alleviated Dox‐induced ferroptosis in a dose‐dependent manner, a phenomenon consistently observed in rat‐derived H9c2 cells (Figure S2J,K). Ferrostatin‐1 (Fer‐1), a potent and selective ferroptosis inhibitor, has been demonstrated to effectively ameliorate DIC [17]. In our in vivo experiments, Mor was administered at doses of 2.5 and 5 mg/kg, while Fer‐1 (1 mg/kg/d) was delivered via intraperitoneal injection for 2 weeks prior to Dox treatment [55]. The results indicated that Mor attenuated Dox‐induced ferroptosis in a dose‐dependent manner, with an efficacy comparable to that of Fer‐1 (Figure S2L,M).

In conclusion, our investigation elucidates Mor's cardioprotective mechanisms against DIC through the lens of ferroptosis inhibition and oxidative stress mitigation. The experimental data demonstrate that Mor exerts its therapeutic effects primarily by the PI3K/AKT/Nrf2/HO‐1 signaling axis. Notably, Mor administration significantly improved survival outcomes and reduced established cardiac injury biomarkers in murine models, underscoring its translational potential for DIC.

## Author Contributions

**Zhi‐Hui Lin:** writing – original draft, writing – review and editing. **Xing‐yu Lin:** visualization, data curation. **Wen‐Jie Lu:** investigation, methodology. **Meng‐Qi Wu:** formal analysis. **Dong‐Yan Song:** funding acquisition, supervision. **Rui‐Dong Cheng:** conceptualization, project administration.

## Conflicts of Interest

The authors declare that no conflicts of interest.

## Supporting information


Supporting File

## Data Availability

The data that support the findings of this study are available from the corresponding author upon reasonable request.
