# Supplementary material for: Morroniside Attenuates Doxorubicin‐Induced Cardiotoxicity by Activating the PI3K/AKT/Nrf2/HO‐1 Pathway to Inhibit Ferroptosis and Oxidative Stress
Source: J Biochem Mol Toxicol. 2026 Jul 27;40(8):e71016. doi: 10.1002/jbt.71016 (PMC13403292; doi:10.1002/jbt.71016)
Supplement: Supplementary file 1 — Supporting File [file JBT-40-e71016-s001.docx]

**Supplement material**

**Table 1**

|  | Reagent | Catalog Number | Source | Country |
| --- | --- | --- | --- | --- |
| 1 | Morroniside | #HY-N0532 | MedChemExpress | USA |
| 2 | RSL3 | #HY-100218A | MedChemExpress | USA |
| 3 | LY294002 | #HY-10108 | MedChemExpress | USA |
| 4 | Doxorubicin | #D1515 | Sigma-Aldrich | USA |
| 5 | Ferrostatin-1 | #SML0583 | Sigma-Aldrich | USA |
| 6 | PTGS2 antibody | #12282 | Cell Signaling Technology | USA |
| 7 | GPX4 antibody | #A11243 | ABclonal | USA |
| 8 | PI3K antibody | #AF6241 | Affinity | USA |
| 9 | p-PI3K antibody | #AF3241 | Affinity | USA |
| 10 | Nrf2 antibody | #12721 | Cell Signaling Technology | USA |
| 11 | Histone- H3 | #A2348 | ABclonal | China |
| 12 | AKT antibody | #10176-2-AP | Proteintech | China |
| 13 | p-AKT antibody | p-AKT | Proteintech | China |
| 14 | HO-1 antibody | #10701-1-AP | Proteintech | China |
| 15 | GAPDH antibody | #10494-1-AP | Proteintech | China |
| 16 | Isoflurane | #R510-22-10 | RWD | China |
| 17 | Cell Counting Kit-8 | #C0038 | Beyotime | China |
| 18 | Superoxide dismutase assay kit | #A001-2-2 | Nanjiang Jiancheng | China |
| 19 | Lactate dehydrogenase kit | #A020-2-2 | Nanjiang Jiancheng | China |
| 20 | Reduced glutathione assay kit | #A006-2-1 | Nanjiang Jiancheng | China |
| 21 | Catalase assay kit | #A007-2-1 | Nanjiang Jiancheng | China |
| 22 | Malondialdehyde assay kit | #A003-2-1 | Nanjiang Jiancheng | China |
| 23 | Creatine kinase-MB isoenzyme assay kit | #E006-1-1 | Nanjiang Jiancheng | China |
| 24 | Cardiac troponin T ELISA Kit | #E-EL-M1801 | Elabscience | China |
| 25 | Dihydroethidium | #S0063 | Beyotime | China |
| 26 | Reactive Oxygen Species Assay Kit | #S0033S | Beyotime | China |
| 27 | Iron Assay Kit | #ab83366 | Abcam | UK |
| 28 | Lipofectamine™ 3000 | #L3000150 | Invitrogen | USA |
| 29 | BCA assay | #P0013B | Beyotime | China |
| 30 | Goat anti-rabbit IgG secondary antibody | #bl003a | Biosharp | China |

**Fig S1.** (A) Assessment of cell viability in H9c2 cells, n = 6. (B-C) Cytotoxicity was detected using cell viability and LDH in all experimental groups, n = 6. (D-E) Quantification of PTGS2 and HO-1 relative fluorescence intensity. (F-G) Quantification of DHE and ROS relative fluorescence intensity. (H) The nuclear translocation of Nrf2 was analyzed by Western blotting in vitro. (I) The levels of Nrf2 were assessed following western blot analysis, n = 6. (J) Immunofluorescence staining was used to detect the nuclear translocation of Nrf2 in all experimental groups, 400x, n = 3. (K) Quantification of PTGS2 relative fluorescence intensity. (L) Assessment of cell viability in all experimental groups, n = 6. (M-N) Quantification of DHE and ROS relative fluorescence intensity. (O-P) Quantification of DHE and ROS relative fluorescence intensity. Data are means ± SD, * ***P*** < 0.05; ** ***P*** < 0.01; *** ***P*** < 0.001.


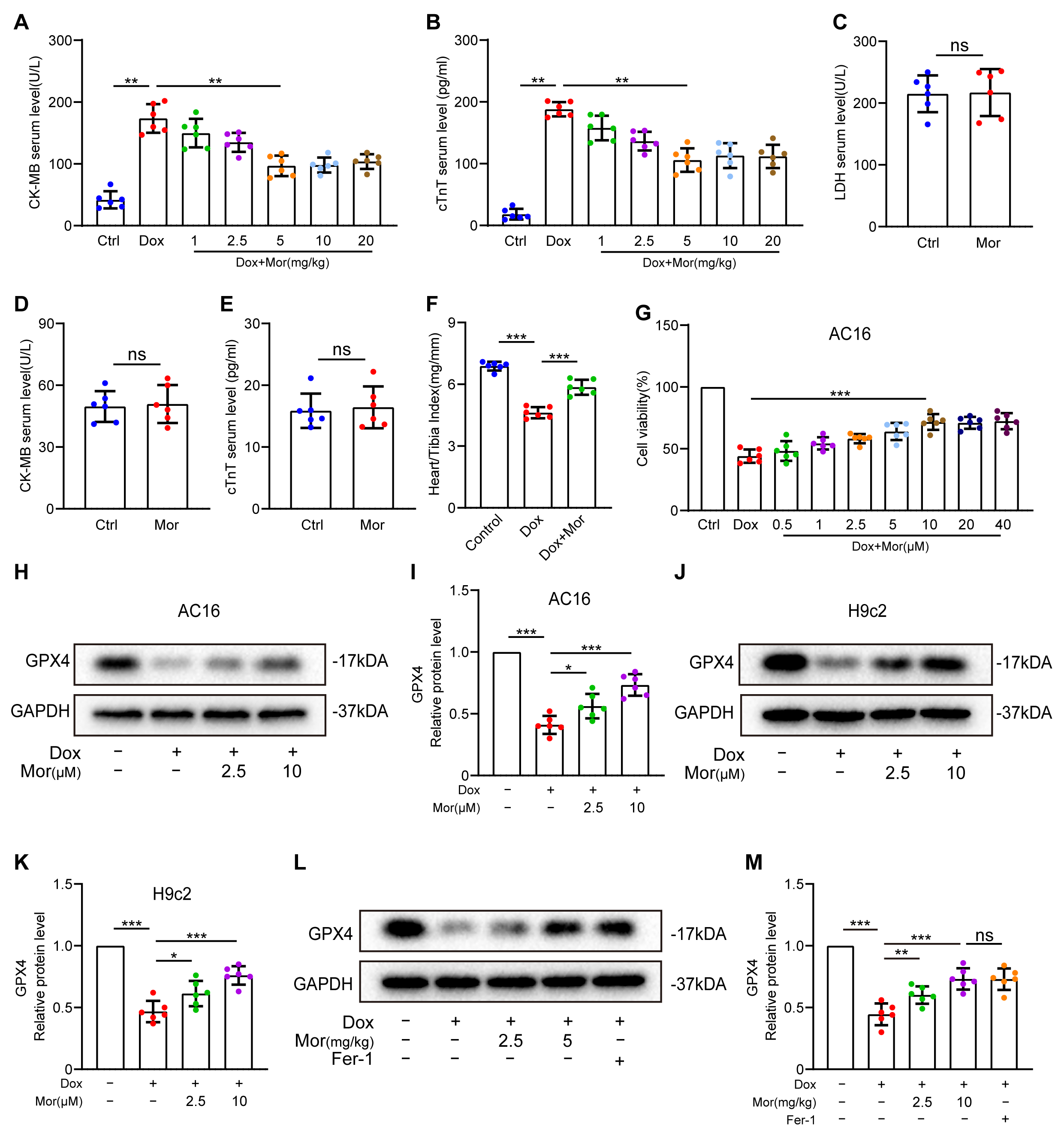


**Fig S2.** (A-B) Assessment of cell viability in all experimental groups, n = 6. (C) We measured the heart weight/tibia length ratio in all experimental mice, n = 6. (D-F) A The concentrations of LDH, CK-MB and cTnT in all experimental groups, n = 6. (G) Assessment of cell viability in AC16 cells, n = 6. (H) Assayed with Western blotting in AC16 cells for GPX4. (I) The levels of GPX4 were assessed following western blot analysis, n = 6. (J) Assayed with Western blotting in H9c2 cells for GPX4. (K) The levels of GPX4 were assessed following western blot analysis, n = 6. (L) Assayed with Western blotting in vivo for GPX4. (M) The levels of GPX4 were assessed following western blot analysis, n = 6. Data are means ± SD, * ***P*** < 0.05; ** ***P*** < 0.01; *** ***P*** < 0.001.
